# Incomplete penetrance of *NOD2* C483W mutation underlining Blau syndrome

**DOI:** 10.1186/s12969-022-00743-1

**Published:** 2022-10-03

**Authors:** Shao-Yu Chang, Naotomo Kambe, Wen-Lang Fan, Jing-Long Huang, Wen-I Lee, Chao-Yi Wu

**Affiliations:** 1 College of Medicine, Chang Gung University, Taoyuan, Taiwan; 2 Department of Dermatology, Kyoto University Graduate School of Medicine, Kyoto, Japan; 3 Genomic Medicine Research Core Laboratory, Chang Gung Memorial Hospital, Taoyuan, Taiwan; 4 Department of Medical Research, Kaohsiung Chang Gung Memorial Hospital, Kaohsiung, Taiwan; 5 Division of Allergy, Asthma, and Rheumatology, Department of Pediatrics, Chang Gung Memorial Hospital, No.5 Fu-Hsing St., Taoyuan, Taiwan, R.O.C.; 6 Department of Pediatrics, New Taipei Municipal TuCheng Hospital, New Taipei City, Taiwan

**Keywords:** Blau syndrome, NOD2, Incomplete penetrance, NFκB

## Abstract

**Background:**

Blau syndrome (BS) is a rare autoinflammatory disorder with *NOD2* gain-of-function mutation and characterized by autoactivation of the NFκB pathway. Classically considered a disease of high penetrance, reports on *NOD2* mutations underlining BS with incomplete penetrance is limited.

**Case presentation:**

The proband is a 9-year-old girl presented with brownish annular infiltrative plaques and symmetric boggy polyarthritis over bilateral wrists and ankles. Her skin biopsy revealed noncaseating granulomas inflammation with multinucleated giant cells. A novel C483W *NOD2* mutation was identify in the proband and her asymptomatic father. Functional examinations including autoactivation of the NFκB pathway demonstrated by *in vitro* HEK293T NOD2 overexpression test as well as intracellular staining of phosphorylated-NFκB in patient’s CD11b^+^ cells were consistent with BS.

**Conclusions:**

We reported a novel C483W *NOD2* mutation underlining BS with incomplete penetrance. Moreover, a phosphorylated-NFκB intracellular staining assay of CD11b^+^ was proposed to assist functional evaluation of NFκB autoactivation in patient with BS.

**Supplementary Information:**

The online version contains supplementary material available at 10.1186/s12969-022-00743-1.

## Background

Blau Syndrome (BS) (OMIM#186,580) is a rare monogenic autoinflammatory disorder also known as juvenile systemic granulomatous disease or early onset sarcoidosis (EOS). Classical triad of dermatitis, polyarthritis, and uveitis are the hallmark manifestations of the disease [1, 2]. Since the identification of *NOD2* mutation as the causative gene underlining BS in 2001 [3], more than 40 pathogenic mutations have been identified in the *NOD2* gene concentrated on or close to the nucleotide-binding oligomerization domain (NOD)/nucleotide-binding and oligomerization (NACHT) subdomain interfaces [2, 4–6]. Generally, BS is believed to inherit in an autosomal dominate fashion with high penetrance [7, 8]. Sporadic cases with indistinguishable clinical features and identical genotypes were referred as EOS [9].

More than 200 cases of BS/EOS have been reported worldwide since its first discovery [5]. While many of the cases presented with its classical triad, variability of clinical features between cases harboring different *NOD2* mutations was noted [10]. Overlapping clinical features of BS/EOS with other inflammatory and granulomatous conditions such as juvenile idiopathic arthritis (JIA), systemic sarcoidosis, anti-neutrophil cytoplasmic autoantibody (ANCA)-associated vasculitis, mycobacterial infection and chronic granulomatous disease (CGD) can sometimes complicate the diagnosis [5, 10, 11]. *NOD2* genetic testing is recommended for the diagnosis of cases suspicious of BS/EOS [9]. However, aside from the common and confirmed variants, functional studies are often required to conclude the cause-and-effect relationship of the mutation and BS/EOS [6, 10, 12].

Here we describes a case of BS/EOS with a novel C483W mutation in the *NOD2* gene. As BS/EOS has previously been considered as a genetic disease with high penetrance, the discovery of asymptomatic carriers in the family harboring the same C483W mutation made the diagnosis challenging. We carefully examine patient’s clinical features, histological and laboratorial data to distinguish BS/EOS from other pathogenic conditions. Nuclear factor kappa B (NFκB) autoactivation of *NOD2* C483W mutation was evaluated utilizing intracellular phosphorylated (p)-NFκB staining and *in vitro* HEK293T NOD2 overexpression test to make the final diagnosis.

## Case presentation

**Fig. 1:**
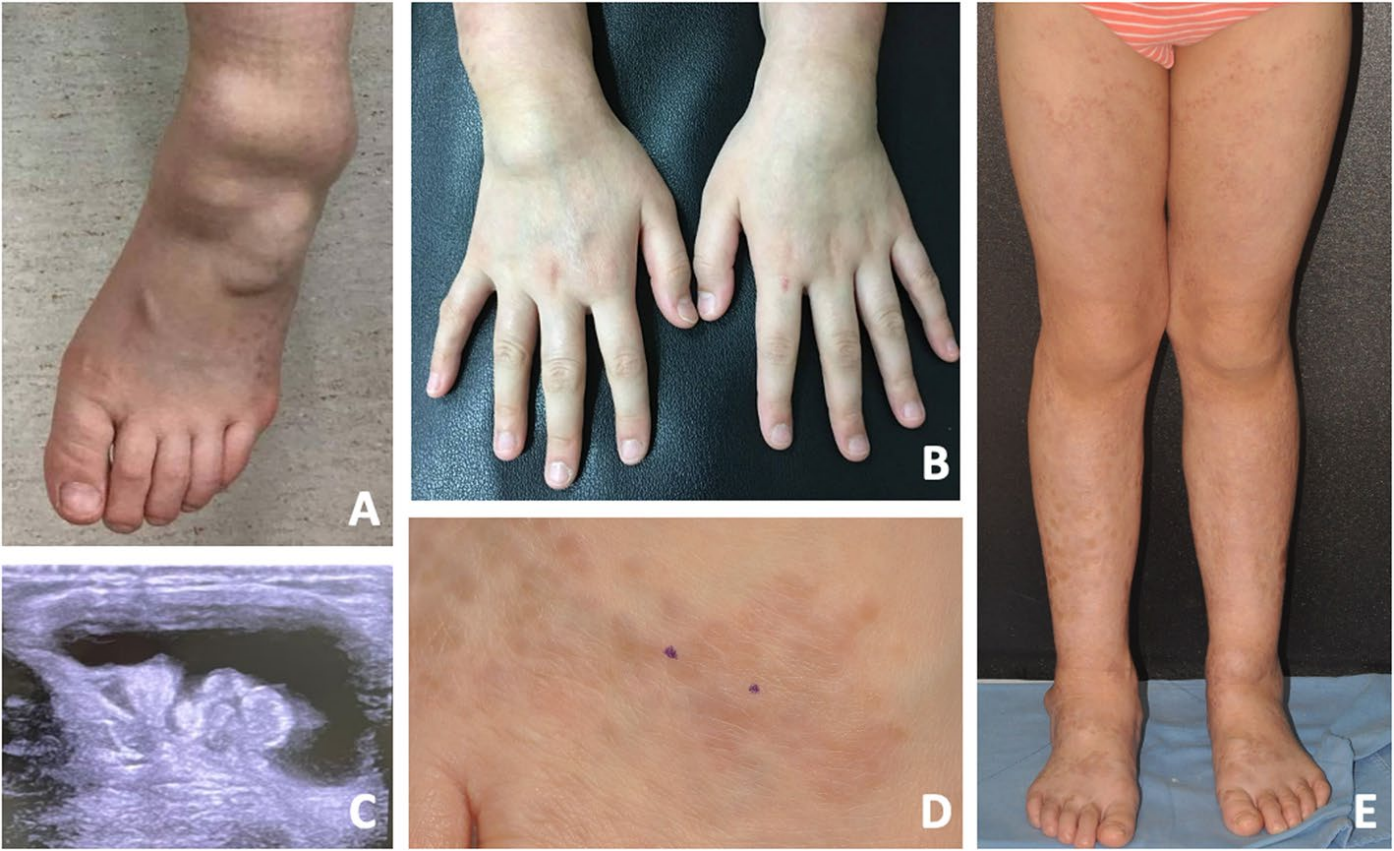
Clinical manifestations of the patient. **A** & **B** Swollen joints over ankles and wrists; **C** Massive para-tendon effusion in proband’s tibial-tarsal joint interface; and **D** & **E** Multiple indurated brownish and violaceous plaques without pruritus or pain over lower legs

A 9-years-old girl was referred to our pediatric rheumatology clinic due to large lumps over bilateral dorsal wrists and ankles since the age of 3 (Fig. 1A and B). No range of motion limitation, camptodactyly or dactylitis was noted during physical examination. While musculoskeletal ultrasound study revealed massive effusion and synovial hyperplasia surrounding extensor tendons in the wrists and para-tendon spaces around bilateral ankle joints (Fig. 1C), the “boggy” joints were disproportionally painless. Evidence of joint effusion and synovial hyperplasia was also noted among many of her proximal inter-phalagneal joints. Despite the chronicity and extravagance of her joint inflammation, the arthritis was non-erosive.

Review her past medical history, non-itchy erythematous skin rash over bilateral legs and forearm suspect of atopic dermatitis was also noted since she was 6-months-old. Emollients and topical steroid were prescribed for the control of her skin lesions with limited effect. Multiple brownish annular infiltrative plaques without pruritus or pain was noted months before her initial visit to our clinic (Fig. 1D and E). No uveitis or intraocular inflammation were recognized during ophthalmic examination. No episodes of prolong fever or fever without source before her visit was recalled by her parents. Vaccinations, including Bacille Calmette-Guerin (BCG) vaccine, was received according to the National Immunization Schedule in Taiwan.

**Fig. 2:**
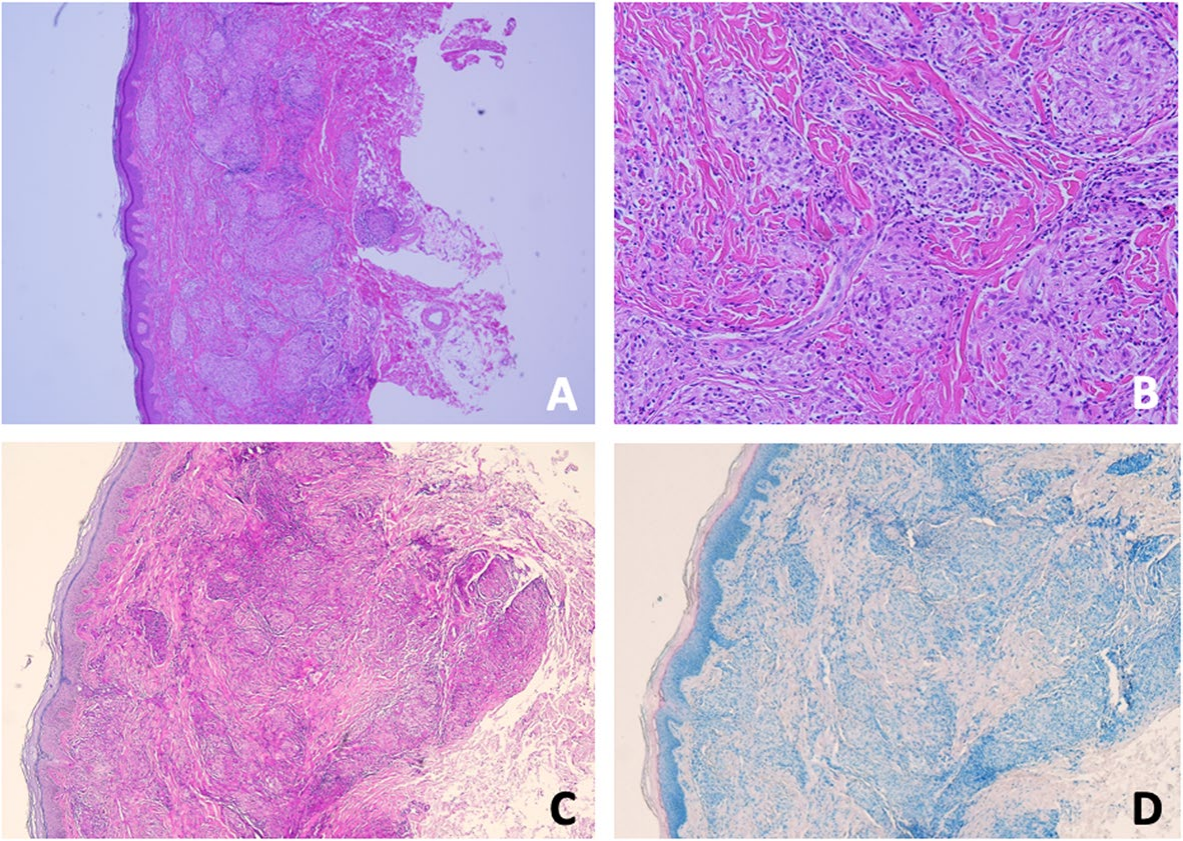
Histopathologic findings and special staining. **A** Hematoxylin and eosin (H&E) stain show hyperkeratosis and heavy infiltrated dermis with multiple small non-caseating granuloma; **B** Occasional multinucleated giant cells and lymphocytes around granulomas; **C** Negative findings under Periodic acid–Schiff stain; and **D** Negative findings under Fite stain

Her serial laboratory tests revealed no leukocytosis (white blood cell count: 8.6 ~ 9.6 × 10^9^ /L), no anemia (hemoglobin level: 11.8 ~ 12.2 g/dL), mild thrombocytosis ( platelet count: 429 ~ 491 × 10^9^ /L), mild elevation of C-reactive protein (5.63 ~ 8.59 g/L; reference level [RR], < 5 g/L) and erythrocyte sedimentation rate (27 ~ 28 mm/h; RR, < 20 mm/h). Levels of complements C3, C4 and tests for antinuclear antibodies, rheumatoid factor, ANCA and HLA-B27 genotype were all negative. Chest radiography study and QuantiFERON-TB Gold test for the survey of intra-thoracic lesions and mycobacterium infection revealed negative results. Skin biopsy from the indurated brownish plaques over her right pre-tibial region revealed small noncaseating granulomas inflammation with multinucleated giant cells and neutrophils infiltration in dermis and subcutis, consistent with sarcoidosis (Fig. 2A and B). Negative findings under acid fast stain, Fite’s stain and Periodic acid–Schiff stain suggested absent of microorganisms and negative polysaccharides accumulation (Fig. 2C and D).

**Fig. 3:**
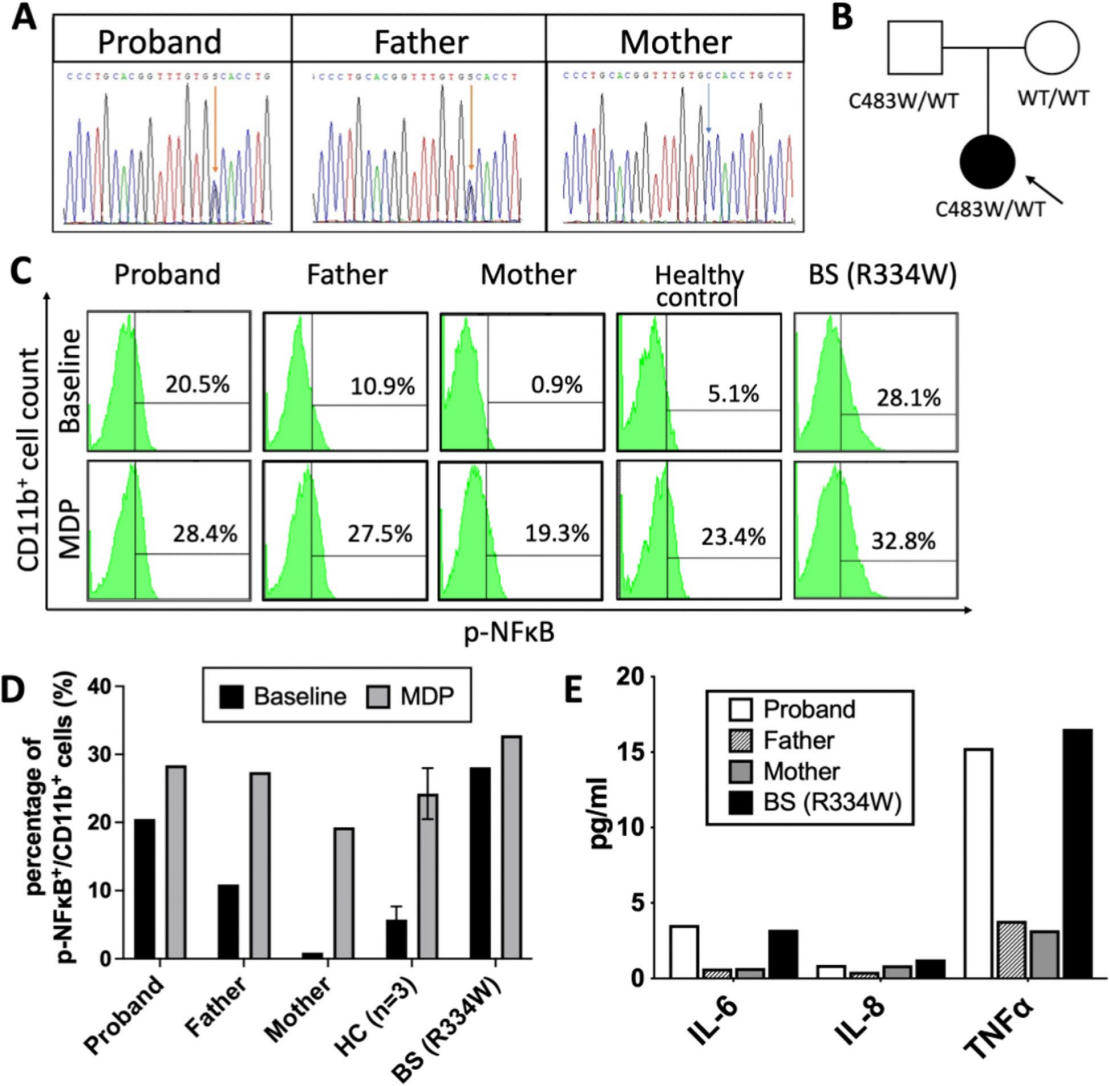
Genotype of the mutant *NOD2* gene and functional assays. **A** The electropherogram shows the sequence of heterozygous c.1449 C > G transition on exon 4 in the NOD/NACHT domain of *NOD2* in the patient and her father (orange arrows). Such mutation was not found in her mother (blue arrow); **B** Family pedigree of the proband; **C** and **D** Representative flowcytometry results and bar graphs depicting the percentage of enhanced p-NFκB staining within CD11b^+^ cells among the proband, her parents, healthy controls (*n* = 3) and a BS patient (R334W). NFκB autoactivation was observed in the CD11b^+^ cells of the symptomatic proband harboring *NOD2* C483W mutant and the BS patients with R334W mutation. **E** Level of cytokines in subjects’ plasma sample

In suspect of Blau syndrome, genomic DNA was extracted from patient’s peripheral blood cells and whole exome sequencing including exon–intron boundaries was performed. A heterozygous c.1449 C > G (NM_022162) mutation on exon 4 in the NOD/NACHT domain of NOD2 on chromosome 16 was identified. To confirm the genotype and clarify its pattern of inheritance, Sanger sequencing of the proband and her parents were further arranged with a result showing that the proband’s mutant was inherited from her father (Fig. 3A and B). No data on the C483W *NOD2* variant was found in published articles or the Infevers database (an online database for autoinflammatory mutations, available at https://infevers.umai-montpellier.fr/) [13, 14]. The functional prediction scores: SIFT = 0.912, PolyPhen2 = 1, CADD = 24 and DANN = 0.994 all asserted the variant as damaging. The allelic frequency was 0 in both the Genome Aggregation Database (https://gnomad.broadinstitute.org/) and the genotype data from the Taiwan Biobank (https://taiwanview.twbiobank.org.tw/) comprised of whole genome sequencing result from 1,475 unrelated healthy individuals in Taiwan. Analysis of the protein variant revealed that the mutation was C483W (p.Cys483Trp).

To evaluate the functional effect of *NOD2* C483W mutant, we isolated peripheral blood mononuclear cells (PBMCs) from the proband, her parents and a BS patient with documented R334W mutation. Cells were washed, Fc receptor-blocked, fixed and permed before staining for intracellular p-NFκB (No.#12–9863-42, eBioscience™) within the CD11b^+^ (No.#15–0118-42, eBioscience™) myeloid cells with and without 100 μg/mL muramyl dipeptide (MDP) stimulation for 30 min. Isotype controls were applied for compensation and cutoff adjustment as summarized in the materials and methods in the Supplementary materials. The p-NFκB^+^/CD11b^+^ cells account for 20.5%, 10.9%, 0.9%, 5.1% and 28.1% of all CD11b^+^ cells in the proband, her father, mother, healthy controls and the BS/EOS control, respectively. In the flowcytometry approach, NFκB autoactivation was observed in CD11b^+^ cells harboring *NOD2* C483W and R334W mutant and further NFκB activation was detected in all samples upon MDP stimulation (Fig. 3C and D). Moreover, likely result from NFκB autoactivation, the level of plasma cytokines IL-6 and tumor necrosis factor-α (TNF-α) were also elevated in the proband and the BS/EOS (R334W) control (Fig. 3E).

**Fig. 4:**
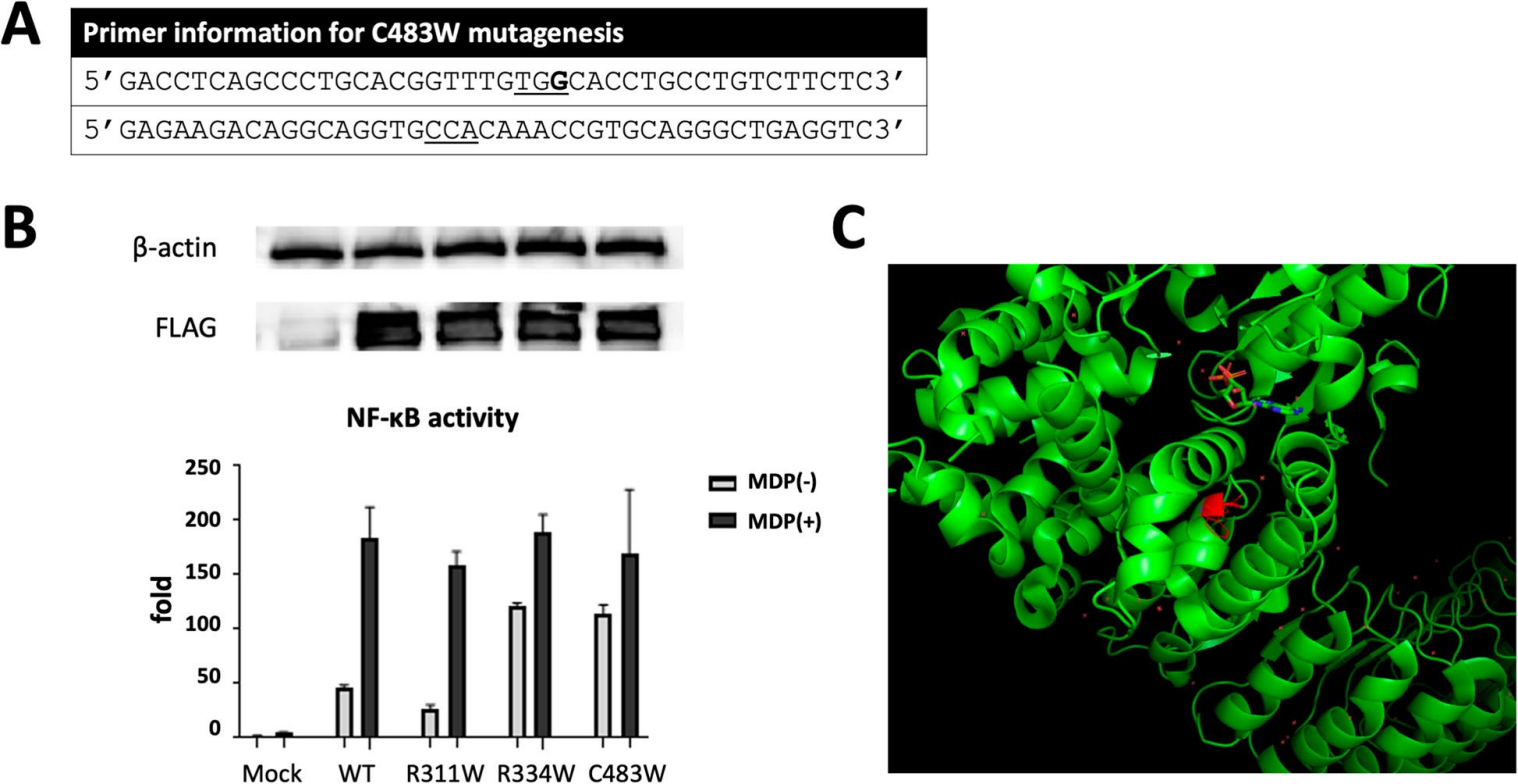
MDP-dependent and -independent NF-κB trans-activation by NOD variants. **A** Primer information for C483W mutagenesis; **B** FLAG for NOD2 expression levels and β-actin analyzed by western blotting are shown in the top column. HEK293T cells transfected with plasmids, containing 100 ng NF-κB reporter plasmid (pNF-κB-Luc), 30 ng expression construct of each human NOD2, 10 ng internal control for normalization of transfection efficiency (pRL-TK), and the corresponding mock vector, were cultured with or without 5 µg/mL MDP for 24 h. NF-κB activity were measured using Duo-Glo Luciferase kit (Promega, #E2920). R334W mutation of NOD2 and R311W SNP were used as positive and negative controls, respectively. Values represent the mean of normalized data (mock without MDP = 1) of triplicate cultures, and error bar indicated SD; and **C** A molecular model of NOD2 was generated and mapped with the position of C483W mutation

Kindly supported by Prof. Naotomo Kambe from the Kyoto University Graduate School of Medicine, Japan, an *in vitro* HEK293T NOD2 overexpression test was performed to evaluate the function of mutant NOD2. In brief, PCR primers for the target mutation, C483W (c.1449C > G), was designed as Fig. 4A. HEK293T cells were seeded and transfected with 1000 ng plasmids, containing 100 ng NFκB reporter plasmid (pNF-κB-Luc), 30 ng expression construct of each human NOD2, 10 ng internal control for normalization of transfection efficiency (pRL-TK), and the corresponding mock vector. The cells were cultured with or without 5 µg/mL MDP for further 24 h and measured for NFκB activity using Duo-Glo Luciferase kit (Promega, #E2920). We used R334W mutation of NOD2 as a positive control and R311W SNP as a negative control. Values represent the mean of normalized data (mock without MDP = 1) of triplicate cultures, and error bar indicated SD. FLAG for NOD2 expression levels and β-actin analyzed by western blotting are also shown in the top column in Fig. 4B. The results supported that the C483W mutation was confirmed to be a gain-of-function mutation. Detail materials and methods were summarized in the Supplementary materials.

According to the patient’s clinical, histological and genetic evidence as well as the NOD2 functional studies, the patient was diagnosed with EOS/BS despite the absence of uveitis during the follow up period. Oral methotrexate at a dose of 10 mg/m^2^/week was prescribed for the control of her arthritis but the swelling and inflammation continues to progress. Additional treatment with 1 mg/kg/day of oral prednisolone greatly mitigated the rashes, but its effect on arthritis control was not satisfying. Adalimumab, a monoclonal antibody against TNF-α, was prescribed 5 months following her initial treatment with noticeable improvement. Regular visits to the ophthalmic clinic was arranged to monitor the development of uveitis.

## Discussions

Here we reported a proband harboring a novel C483W mutation in the *NOD2* gene with wide spread brownish plaques, symmetric “boggy” arthritis and non-caseating granulomas in the skin specimen suspicious of BS/EOS. Through genetic testing and functional exams, our data supported the pathogenicity of C483W *NOD2* mutation underlining BS/EOS with incomplete penetrance. Moreover, we proposed a novel assay utilizing intracellular p-NFκB staining of CD11b^+^ cells to functionally evaluate the autoactivation of NFκB in patient with BS/EOS.

### Clinical manifestations

As a prototypic autoinflammatory granulomatous disease, dermatitis, polyarthritis, and uveitis are the classical triad of BS/EOS with rash being the first feature [1, 2, 15]. Usually painless and non-pruritic, non-caseating granulomas is the typical findings seen in skin biopsy of BS/EOS and our index case [5]. This is different from the caseating granulomas seen in CGD, ANCA-associated vasculitis and granuloma forming infections, such as tuberculosis, leprosy, atypical mycobacteria, or fungal infection [16]. Moreover, Crohn’s disease (CD) and sarcoidosis are also inflammatory syndromes characterized with non-caseating granuloma involving many organ systems [2, 5, 8]. In contrast to the simple granulomas seen in the biopsy specimen from patients with CD, the histopathogenical features of BS usually demonstrated polycyclic granulomas with large lymphocytic coronas and extensive emperipolesis of lymphocytes within multinucleated giant cells, accompany fibrinoid necrosis and fibrosis [17]. Classical sarcoidosis mostly affect young adults 30–50 years of age [8]. BS/EOS, on the other hands, are usually found in children harboring *NOD2* mutation before the age of 5 with dominate extra-thoracic manifestations and less lymph node involvement [5].

Arthritis is a dominate feature seen in the proband and the most common manifestation presenting in over 90% of all BS/EOS patients [5, 18]. Although camptodactyly, the digital flexion deformity seen in half of BS/EOS cases, was not observed in the proband, extensive polyarthritis with “boggy” appearance involving wrists, knees, ankles and proximal interphalangeal joints in symmetry is compatible with most reported case diagnosed with BS/EOS [15, 18]. Similar to that in polyarticular JIA and enthesitis related arthritis, excess joint swelling and tenosynovitis with massive joint effusion can be seen in both large and small joints in symmetry [19]. However, the absent or subtle raise of acute phase reactants, lack of joint pain and joint destruction, and relatively well preserved range of motion in the large joints are more commonly observed in patients with BS/EOS and our proband [15]. Non-caseating granulomas in the synovial specimen are also in favor of BS/EOS [17, 20].

Uveitis is usually the latest feature presented in BS/EOS. It can lead to visual loss and is the most concerned morbidity of the disease [21]. Because BS/EOS associated uveitis mostly develops after a median disease duration of 12.1 years, it may not be observed early in the disease course, such as our proband [18]. Uveitis has been reported to affect up to 75% of the patients suffering BS/EOS and is predominately bilateral. Compared to the uveitis of JIA which is almost always anterior, panuveitis with typical multifocal choroidal scars is the most observed feature in BS/EOS uveitis [20, 21]. Optic disc abnormalities, band keratopathy, cataract, glaucoma, retinal vasculitis, and macular edema are ocular complications which have also been reported [20, 21]. Fortunately, the use of biologics, particularly TNF-α targeting monoclonal antibodies, have been shown to mitigate ocular inflammation and prevent blindness to a certain degree [8, 21]. Despite appropriate treatment, however, visual prognosis in Blau syndrome remains guarded.

### Genetic analysis

While BS/EOS patients without *NOD2* mutations were reported, mutations in the *NOD2* gene is perhaps the most apparent difference distinguishing BS/EOS from other granulomatous conditions [5]. Unlike systemic sarcoidosis, JIA, CGD, ANCA-associated vasculitis and granuloma forming infections, *NOD2* mutation is associated with chronic inflammatory disorders such as BS/EOS and CD [2, 4]. Accounting for approximal half of all reported BS/EOS, heterozygous missense mutations of R334W and R334Q are the most common disease causing mutations [10, 15, 18]. Recently, *Maekawa* et al. analyzed the crystal structure of NOD2 protein and revealed that most of the BS/EOS associated gain-of-function *NOD2* mutations located in the NOD/NACHT domain interface surrounding the magnesium and adenosine triphosphate binding sites or concentrated on the helical domain 1 (HD1) [4, 6]. According to the structural analysis and its proinflammation nature, the BS/EOS associated mutations are likely to interfere NOD2 inner domain interactions and promote conformational changes to its bioactive form [4]. On the other hands, CD associated mutations are widely distributed throughout the protein [4]. Three variants within leucine rich repeat domains of the *NOD2* gene, R702T, G908R and L1007fsinsC were identified as susceptibility loci associated with CD [2]. Unlike BS/EOS, these mutations have been postulated to disrupt the formation of oligomer or binding of its ligands, which result in defective NFκB activation, reduced α-defensin synthesis and altered intestinal microbiome [2, 4]. The C483W mutation discovered in our proband and her father, is located on the HD1 within the NOD/NACHT domain, clustered with the BS/EOS associated variants. According to Maekawa’s work [4], the positioning and molecular changes of C483W may potentially destabilized the closed form of NOD2 and promote conformational change from the inactive to the active form, leading to constitutive activation of the protein.

### Functional analysis

Physiologically, NOD2 protein directly recognizes intracellular bacterial fragments containing the MDP motif. Ligand interaction frees intra-molecular autoinhibitory conformation, leading to NOD2 oligomerization. Through caspase recruitment domain (CARD)-CARD interactions, subsequent activation of the NFκB and mitogen-activated protein kinase pathways results in the up-regulated transcriptions of pro-inflammatory and host defense genes [4, 6, 12, 22]. Specifically, many studies demonstrated that BS/EOS associated gain-of-function *NOD2* mutations result in NFκB autoactivation and subsequently lead to overexpression of cytokines involved in the auto-inflammatory process [4, 6, 10, 15, 23, 24]. Impaired NOD2 activation to MDP resulted in mitigated NFκB signaling and absence of spontaneous proinflammatory cytokine production have also been reported by others [12, 24, 25].

NFκB autoactivation in BS/EOS have been demonstrated in various ways. An *in vitro* NFκB luciferase reporter system with overexpression of mutant *NOD2* in the HEK293T cells, as demonstrated in Fig. 4B, is perhaps the most commonly performed test to functionally examine the impact of BS/EOS *NOD2* mutations in real world practice [6, 10, 23]. However, due to unavoidable limitations of the artificial system in cell line experiments, these assays may not reflex the true physiologically alteration of the immune responses in BS/EOS. For example, HEK293T cell line may not contain the complete endogenous regulatory elements for NOD2 and the transfection of mutated *NOD2* plasmid may likely mimic homozygotic *NOD2* mutation. To elucidate the mechanisms of autoinflammation in patients with BS/EOS and to precisely evaluate the immune phenotypes, *Takada* et al. established a BS specific induced pluripotent stem cell (iPSC) line from a BS patient and applied the CRISPR-Cas9 system to correct the disease-associated *NOD2* mutation [24]. Utilizing both an NFκB luciferase reporter assay and staining for intracellular NFκB p65, autoactivation of NFκB pathway was clearly demonstrated in human samples with mutant *NOD2* [24]. However, to establish a BS specific iPSC line for each *NOD2* mutation is not clinically practical considering its technical difficulty. Known that NOD2 is mainly expressed in hematopoietic lineage cells, particularly in monocytic cells [26], we gated on the CD11b^+^ PBMCs to evaluate their NFκB activity in subjects harboring wide type and mutant NOD2. Similar to *Takada’s* finding examine intracellular staining of NFκB p65 in *NOD2*-mutated immortalized proliferating myeloid cell lines with confocal microscope [24], the percentage of intracellular p-NFκB staining in the MDP treated CD11b^+^ cells were generally increased regardless of underlining *NOD2* genotypes. The shifting of p-NFκB staining without MDP stimulation, however, was elevated only in confirmed BS control and the symptomatic proband harboring C483W *NOD2* mutations. Extensive testing on other known BS/EOS *NOD2* mutations is warranted to uphold this novel assay in assisting the diagnosis of BS/EOS functionally.

### Incomplete penetrance

To date, E383K is the only confirmed *NOD2* mutation underlining BS/EOS with incomplete penetrance [7, 10]. Due to the shortage of genetic material from elder family members of the proband, we were unable to confirmed the pattern of penetrance via classical segregation study. However, the fact that proband’s father harbors the same C483W *NOD2* mutation but lacks clinical BS/EOS phenotype itself suggests that this *NOD2* mutation was incompletely penetrated.

While the exact regulatory network of NOD2 and its impact in disease development requires further elucidation, the functional assays including the CD11b^+^ intracellular p-NFκB staining experiment and the plasma cytokine profile seem to closely associate with subjects’ clinical manifestations despite their *NOD2* genotypes. Specifically, the percentage of p-NFκB^+^/CD11b^+^ cells and the plasma level of proinflammatory cytokines seem to be lower in proband’s father as compared to the proband and the positive BS control (Fig. 3C, D and E). Our immune system is made up with a complex and dynamic biological web of molecular, cellular, and organismal networks. The existence of promoting or protective factors may likely alter the development of the inflammation and clinical phenotype. For example, NOD2 interacts with receptor-interacting protein 2 as well as transforming growth factor β-activated kinase 1 and gene associated with retinoid-IFN-induced mortality 19 to mediate NFκB activation in cells [27]. Caspase-associated recruitment domain 12 negatively regulates NOD-protein-mediated NFκB activation through a NOD-NOD interaction and Erbin, a member of the PDZ domain-containing family, is known to inhibit NOD2 and capable of altering cytokine expression in response to MDP stimulation [27, 28]. Genetic and extrinsic factors interacting with this network of elements can potentially alter the immune responses and direct clinical manifestation beyond NOD2 mutation itself. Moreover, TNF-α, IFN-γ and toll-like receptor ligands were known priming triggers to promote NOD2 expression [24, 29, 30]. Environmental stimuli such as BCG vaccination or Propionibacterium acnes infection have also been reported to trigger the development of BS/EOS [8, 20, 24]. While the exact regulatory mechanism requires further elucidation, data from our p-NFκB intracellular staining assay and the level of plasma cytokines suggested that there likely exists other factors modulating the immune regulation in the asymptomatic subject harboring C483W *NOD2* mutation.

## Conclusions

In conclusions, we’ve demonstrated that C483W *NOD2* mutation is a novel mutation underlining BS/EOS with incomplete penetrance. In addition, we proposed a p-NFκB intracellular staining assay to potentially assist functional evaluation of BS/EOS associating NODs mutations.

## Supplementary Information

**Additional file 1: Supplementary materials.**

## Data Availability

The datasets used and/or analyzed during the current study are available from the corresponding author on reasonable request.

## Abbreviations

ANCA: Anti-neutrophil cytoplasmic autoantibody
BCG: Bacille Calmette-Guerin
BS: Blau syndrome
CARD: Caspase recruitment domain
CD: Crohn’s disease
CGD: Chronic granulomatous disease
EOS: Early onset sarcoidosis
DH1: Helical domain 1
IL: Interleukin
iPSC: Induced pluripotent stem cell
JIA: Juvenile idiopathic arthritis
MDMs: Monocyte-derived macrophages
MDP: Muramyl dipeptide
NACHT: Nucleotide-binding and oligomerization
NFκB: Nuclear factor kappa B
NOD: Nucleotide-binding oligomerization domain
p: Phosphorylated
PBMCs: Peripheral blood mononuclear cells
TNF-α: Tumor-necrosis factor-α
