# Supplementary material for: Incomplete penetrance of NOD2 C483W mutation underlining Blau syndrome
Source: Pediatr Rheumatol Online J. 2022 Oct 3;20:86. doi: 10.1186/s12969-022-00743-1 (PMC9531522; doi:10.1186/s12969-022-00743-1)
Supplement: Supplementary file 1 — Additional file 1: Supplementary materials. [file 12969_2022_743_MOESM1_ESM.docx]

**Supplementary materials**

1. **Intracellular staining of phosphorylated-NFκB in CD11b^+^ cells**

*Muramyl dipeptide (MDP) stimulation test*

After isolating human peripheral blood mononuclear cells (PBMCs) via Ficoll-Paque gradient centrifugation, cells were counted and diluted to the concentration of 1x10^6^ cells/ml in culture medium. The cells were stimulated with or without MDP 100 µg/ml (No.#A9519, Sigma) for 30 mins before fixed with equal amount of pre-warmed Fix Buffer (No. #557870, BP Phosflow^TM^) in a 37°C water bath for 10 mins. Cells were spin down at 250 x g for 10 min, discarded the supernatants and washed with Stain buffer (No. #554656, BP Pharmingen^TM^) once.

*Intracellular staining of p-NFκB*

1 µg/tube of Fc receptor binding inhibitor (No.#14-9161-73, eBioscience^TM^) were added in the washed PBMCs and left in 4°C fridge for 15 mins before washed with Stain buffer. CD11b-PECy5 (No.#15-0118-42, eBioscience^TM^) were added to the tubes and left in 4°C fridge for 30 mins before the cells were again washed with Stain buffer. Cells were permeabilized with 100 µg/tube of pre-cooled Perm III buffer (No. #558050, BP Phosflow^TM^) on ice for 30 mins then washed twice with Stain buffer. p-NFκB-p65 Ab-PE (No.#12-9863-42, eBioscience^TM^) and isotype control (eBM2a) (No.#12-4724-82, eBioscience^TM^) were added to the tubes and left in 4°C fridge for 30 mins before the cells were again washed with Stain buffer. Cells were washed and diluted to proper amount for flowcytometry analysis. Live CD11b cells were gated and the cutoff for p-NFκB was adjusted according to the isotype controls result.

**B. In vitro HEK293T NOD2 overexpression test:**

*C483W mutagenesis*

PCR primers for the target mutation, p.C483W (c.1449C>G), was designed as **Figure 4A**. PCR was performed with the high-fidelity enzyme, KOD plus (TOYOBO, #KOD-211). After *Dpn I* (Takara, #1235A) digestion to destroy E coli-derived templates, the PCR constructs were transformed into Jim109 competent cells (Nippon Gene, #313-06243). After mini-prep, we checked the sequence of PCR amplified region, then cut with *Hind III* of multi-cloning sites of the plasmid (p3xFLAG-CMV, Sigma-Aldrich, #E7908) and *EcoR I* in the wild type of human NOD2, and swapped the PCR amplified region into the plasmid containing human NOD2.

*Luciferase assay*

HEK293T cells were seeded at a density of 1.0 × 10⁵/well on a sterile 24 well plate. After 24 hours, cells were transfected with 1000 ng plasmids, containing 100 ng NF-κB reporter plasmid (pNF-κB-Luc), 30 ng expression construct of each human NOD2, 10 ng internal control for normalization of transfection efficiency (pRL-TK), and the corresponding mock vector, using TransIT-293 Transfection Reagent (Mirus Bio, #MIR2700). The cells were cultured with or without 5 µg/mL muramyl dipeptide (MDP; InvivoGen, #tlrl-mdp) for further 24 hours and measured for NF-κB activity using Duo-Glo Luciferase kit (Promega, #E2920). We used p.R334W mutation of NOD2 as a positive control and p.R311W SNP as a negative control. Values represent the mean of normalized data (mock without MDP=1) of triplicate cultures, and error bar indicated SD.

FLAG for NOD2 expression levels and β-actin analyzed by western blotting are also shown in the top column of **Figure 4B**. For the 1^st^ antibody, anti-β-actin (clone 6D1, MBL, #177-3) and anti-FLAG (clone FLA-1, MBL, #185-3B) was incubated for 20 min and then HRP-conjugated horse-derived anti-mouse IgG (Vector, #PI-2000) was incubated for 15 min. The membrane was exposed with ECL.
